# Everybody's Page

**Published:** 1892-06-25

**Authors:** 


### EVERYBODY'S PAGE.

A Masonic lodge has been established in Paris under the title of the "Lodge of Scientific Materialism." The Materialist of the present day is generally at the best but an iconoclast, who has no originality, and his reasonings are often more remarkable for dogmatism than for scientific accuracy. All this will no doubt be altered by the new society, which has had its birth appropriately in the home of Materialism.

---

An American newspaper offers a very sensible suggestion to the belligerent statesmen of Brazil, to the effect that they should turn their warlike energies into a more profitable channel, and begin a campaign against the yellow fever. This scourge has claimed so many victims and desolated so large a tract of that country that it would prove a worthy enemy to try issue with. But it seems hopeless to expect South American Republics to give attention to anything so useful and beneficial to the citizens, for whose good they are supposed to be established, as sanitary regulations. Yellow fever is one of those preventable diseases which seem to be inflicted upon man as a punishment for wilful neglect.

---

Women intend to take a prominent place in the forthcoming World's Fair, and the "Woman's Building" is sure to attract much attention. Leading manufacturers throughout the States have given every encouragement to women in their employ to prepare exhibits which shall be entirely their own work from design to completion. In the Washington State exhibit woman's work will be a very conspicuous feature, and the women of Missouri are also going to assert themselves. A Woman's Dormitory Association has been formed, the object of which is to furnish cheap and comfortable living quarters to women visitors while the exhibition is open. The association, if its four huge hotels are well managed, will prove a great success and boon.

---

Those who use morphine should take warning from the fate of the novelist, Guy de Maupassant, and those who are curious as to the effects of this drug on the human system will find their curiosity satisfied by reading his novel "Horla," in which they will find a vivid description of its action.

---

Cows in Norway have been credited with the power of exacting sustenance from stones which travellers in that country will have seem them licking. A more substantial diet is found for them in the winter in some parts of the country, where provender of all kinds is scarce, in the shape of heads of the cod-fish which are pounded into a mash. The milk of cows so fed tastes strongly of cod-liver oil and cannot be pleasant to drink, yet with all its unpleasantness it may be very valuable for consumptive patients, though we are not aware it has been put to such a use.

---

So long is the list of preventable diseases that their complete suppression seems an Augæan labour beyond the powers of any modern Hercules. Yet their suppression, so far as science is concerned, is far from being an impossible achievement, though unfortunately it seems a highly improbable one. The grea barrier in the way is apathy, and occasionally ignorance. However systematically disinfection may be carried out, the best results cannot possibly be attained without establishing perfect isolation. And this latter precaution, really so essential, is far too frequently neglected. Our faith in some disinfectants, hitherto considered reliable, has recently received some rude shocks. Sulphur, according to some authorities, possesses a reputation beyond its deserts, and, notwithstanding its odour, which is suggestive of much power for destructiveness, is deficient in effectiveness. As aids to it, solutions of mercuric-chloride and carbolic acid, and chloride of lime are suggested.

